# CD4^+^ CD25^+^ FOXP3^+^ T cell frequency in the peripheral blood is a biomarker that distinguishes intestinal tuberculosis from Crohn’s disease

**DOI:** 10.1371/journal.pone.0193433

**Published:** 2018-02-28

**Authors:** Veena Tiwari, Saurabh Kedia, Sushil Kumar Garg, Ritika Rampal, V. Pratap Mouli, Anuja Purwar, D. K. Mitra, Prasenjit Das, S. Dattagupta, Govind Makharia, S. K. Acharya, Vineet Ahuja

**Affiliations:** 1 Department of Gastroenterology and Human Nutrition, All India Institute of Medical Sciences, New Delhi, India; 2 Department of HLA and Transplant Immunology, All India Institute of Medical Sciences, New Delhi, India; 3 Department of Pathology, All India Institute of Medical Sciences, New Delhi, India; University of South Carolina School of Medicine, UNITED STATES

## Abstract

**Background:**

Distinguishing between Crohn’s Disease (CD) and Intestinal Tuberculosis (ITB) has been a challenging task for clinicians due to their similar presentation. CD4^+^FOXP3^+^ T regulatory cells (Tregs) have been reported to be increased in patients with pulmonary tuberculosis. However, there is no such data available in ITB. The aim of this study was to investigate the differential expression of FOXP3^+^ T cells in patients with ITB and CD and its utility as a biomarker.

**Methods:**

The study prospectively recruited 124 patients with CD, ITB and controls: ulcerative colitis (UC) and patients with only haemorrhoidal bleed. Frequency of CD4^+^CD25^+^FOXP3^+^ Tregs in peripheral blood (flow cytometry), FOXP3 mRNA expression in blood and colonic mucosa (qPCR) and FOXP3^+^ T cells in colonic mucosa (immunohistochemistry) were compared between controls, CD and ITB patients.

**Results:**

Frequency of CD4^+^CD25^+^FOXP3^+^ Treg cells in peripheral blood was significantly increased in ITB as compared to CD. Similarly, significant increase in FOXP3^+^ T cells and FOXP3 mRNA expression was observed in colonic mucosa of ITB as compared to CD. ROC curve showed that a value of >32.5% for FOXP3^+^ cells in peripheral blood could differentiate between CD and ITB with a sensitivity of 75% and a specificity of 90.6%.

**Conclusion:**

Phenotypic enumeration of peripheral CD4^+^CD25^+^FOXP3^+^ Treg cells can be used as a non-invasive biomarker in clinics with a high diagnostic accuracy to differentiate between ITB and CD in regions where TB is endemic.

## Introduction

Intestinal tuberculosis (ITB) and Crohn’s disease (CD) are chronic granulomatous disorders with clinical, radiological and endoscopic similarities that make the differentiation between these two conditions a challenging as well as a perplexing task [1–5]. In 1913, Dalziel first noted resemblances between CD and gastro-intestinal tuberculosis [6]. It has almost been a century from the first description of the similarity but still the debate of differentiation between CD and ITB continues and no confirmatory modality is available. CD no longer remains the disease of industrially developed countries, as its incidence is on rise in Asian countries [7–9]. On the other hand, globalization and the HIV pandemic has also caused a surge in incidence of TB and consequently abdominal TB in the western world. The changing epidemiology of CD and ITB over the last decade has caused the diagnostic dilemma between the two conditions to become more evident.

Currently, response to anti tubercular therapy (ATT) is a useful therapy to differentiate ITB from CD [10]. However, this leads to a time lag for a definite diagnosis, and hence there is a significant delay in diagnosis and initiation of therapy. A misdiagnosis of ITB patient as CD, can expose the patient to the detrimental effects of the steroids. Therefore, a non-invasive test (biomarker) is required to differentiate between these two modalities to save the time required to make a proper diagnosis.

Under steady state, the intestinal mucosa maintains an equilibrium between inflammation to pathogenic bacteria and tolerance to self-antigen and commensal bacteria. Functionally specialized subsets of CD4^+^ T cells play a key role in the regulation of mucosal immune responses. One such subset is T regulatory cells (Tregs), defined by the expression of CD4, CD25 and the transcription factor forkhead box P3 (FOXP3) which plays an influential role for its development and function. Tregs have been reported to sustain immunological tolerance while limiting the inflammatory immune response, thereby maintaining immunological homeostasis. During inflammation, FOXP3^+^ T regulatory cells have been reported to be expanded and accumulated in the inflamed mucosa of IBD patients [11,12]. Recently, many studies have also shown an increase in CD4^+^CD25^+^FOXP3^+^ T lymphocytes in the blood and at the site of active infection in pulmonary tuberculosis patients [13–15].

However, there is no data which depicts the role of FOXP3^+^ T regulatory cells in ITB patients. Therefore, we hypothesized that there might be differences in Treg frequencies in an aberrant immune response disease like CD and an infectious disease like ITB. For validating our hypothesis, we did a pilot study and found that the FOXP3 mRNA expression was significantly elevated in colonic biopsies obtained from ITB patients as compared to patients with CD, suggesting that FOXP3 mRNA expression in colonic mucosa could be a discriminatory marker between ITB and CD [16]. Hence, the present study with an increased sample size was planned to investigate the comparative frequency of FOXP3^+^ T cells in patients with ITB and CD in peripheral blood and in colonic mucosa (disease site), and to further investigate its utility as a biomarker to differentiate between these two diseases.

## Materials and methods

### Patient population

A single center prospective cohort study was conducted at Inflammatory Bowel Disease clinic at All India Institute of Medical Sciences, New Delhi, India. The study population comprised of ITB and CD patients; in addition to ulcerative colitis (UC) patients, which were included as disease controls. Adult patients with suspected haemorrhoidal bleed and planned for sigmoidoscopy were also recruited as controls. CD and ITB patients with less than 18 years of age, isolated small bowel involvement, pregnant or lactating, with co-existing infection (pulmonary, urinary), HIV seropositivity, associated malignancy, and not ready for long term follow up, were excluded from the study. The Ethics committee, All India Institute of Medical Sciences, New Delhi, approved the experimental protocols and all the methods carried out in the study were according to the institutional guidelines (Ref. No. T-3/28.11.2008). Written informed consent was obtained from control and patient population prior to their recruitment according to the institutional guidelines.

### Definitions

#### Crohn’s disease

The patients were diagnosed as CD based on the European Crohn’s and Colitis Organization (ECCO) guidelines [17], with a combination of clinical, endoscopic and histological features. Only patients with ileocolonic disease were included in this study to maintain the homogeneity of disease location. Disease activity was assessed by Crohn’s Disease Activity Index (CDAI) and patients with CDAI < 150 were considered to be in remission [18].

#### Intestinal tuberculosis

The diagnosis of ITB was made on the basis of characteristic clinical features (abdominal pain, constipation and / or diarrhea, constitutional symptoms, and intestinal obstruction), endoscopic features (ileocecal area involvement, ulcerations, nodularity, and strictures), histological features (presence of caseating granulomas) and microbiological tests (presence of acid-fast bacilli on the smear examination or culture), and response to ATT (Paustian’s criteria with Logan’s modification) [19,20]. Only patients with ileocolonic tuberculosis were included to maintain the homogeneity of disease location.

#### Ulcerative colitis

The diagnosis of UC was made on the basis of the European Crohn’s and Colitis Organization (ECCO) consensus statement [21]. Disease activity was assessed by Mayo Score and patients with Mayo score < 3 were considered to be in remission [18].

#### Controls

Patients with suspected haemorrhoidal bleed, taken up for diagnostic sigmoidoscopy were included as controls.

### Sample collection

10 ml of peripheral blood was taken from controls, CD, ITB (before antitubercular therapy), and UC patients in ethylene diamine tetraacetic acid (EDTA) vacutainer for RNA isolation and heparinized vacutainers for peripheral blood mononuclear cells (PBMCs) isolation. 6–8 mucosal tissues were taken during colonoscopy (patients) and sigmoidoscopy (controls) in 10% formalin for immunohistochemistry and RNA isolation.

#### Immunophenotyping by flowcytometry

Peripheral venous blood was obtained from controls and patient (CD, UC and ITB) population after written informed consent. Peripheral blood mononuclear cells (PBMCs) were isolated by Ficoll density gradient centrifugation and stained for surface markers using anti-CD4 phycoerithrin (PE), anti-CD25 allophycocyanin (APC) and anti-CD127 phycoerithrin Cy7(PE-Cy7) for 30 minutes at room temperature (BD Biosciences, San Diego, USA). Subsequently, the cells were fixed, permeabilized with 1X permeabilisation buffer (eBioscience, CA, USA), followed by intracellular staining with anti-FoxP3 antibodies (eBiosciences, CA, USA) for 30 minutes at 4°C. Data were acquired on CyAn (Beckman Coulter, Germany) and analysed using SUMMIT V4.3 software. Results are represented as mean percentage of FOXP3 positive cells gated on CD4^+^CD25^+^CD127^-^ cells.

#### Double immunostaining

**Fig 1:**
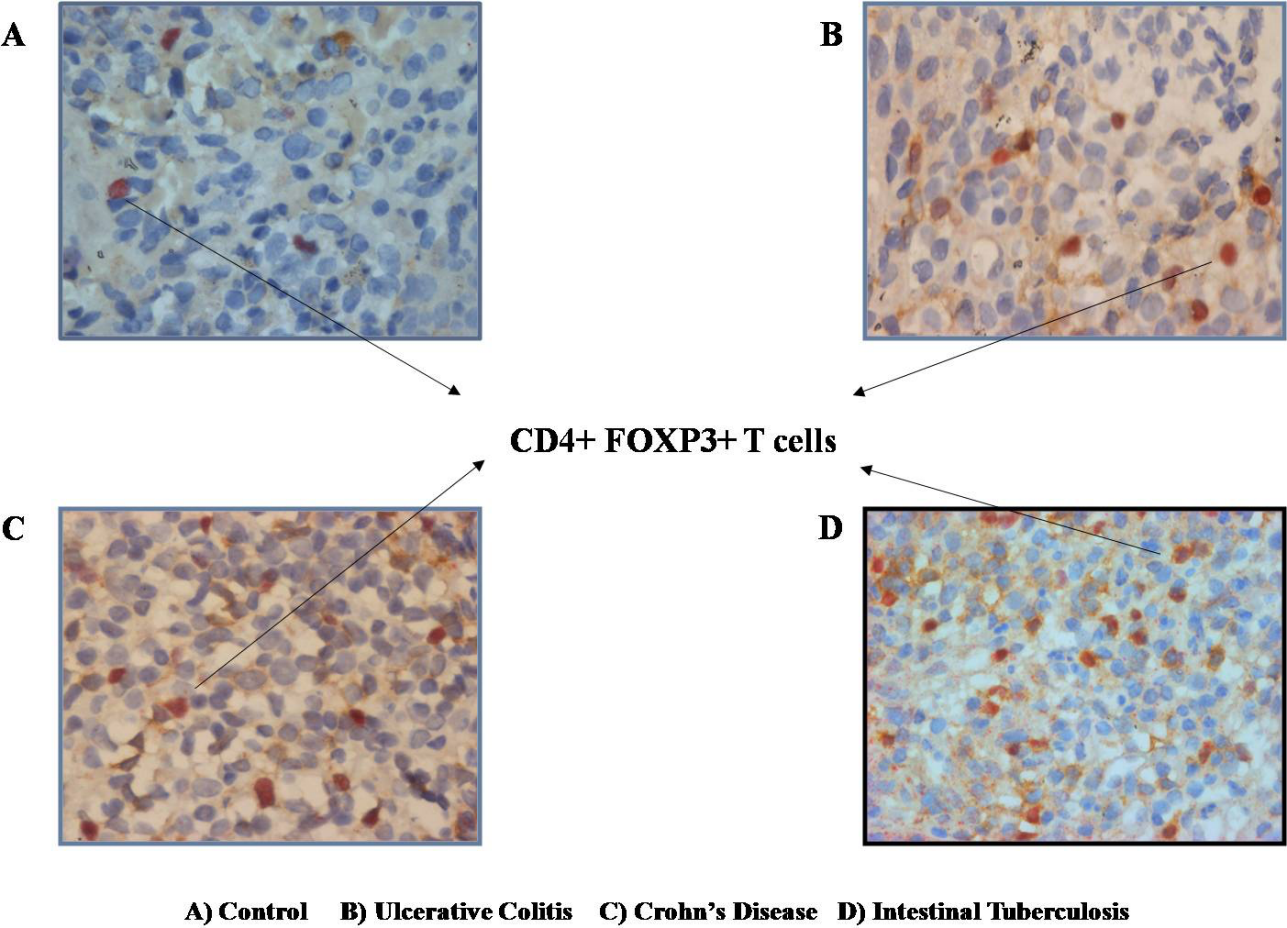
Representative photomicrographs (40X objective at 400 magnifications) of colonic biopsy showing increased frequency of CD4^+^FOXP3^+^ dual positive cells in all diseased groups as compared to control: a) control, b) ulcerative colitis, c) Crohn’s disease, d) intestinal tuberculosis. Scale: 40X objective; 400 magnification.

5 μm thick sections from paraffin blocks of intestinal tissue and tissue of tonsil (as positive control) were first treated with rabbit monoclonal antihuman CD4 antibody (Spring Bioscience), followed by mouse anti-Human FOXP3 monoclonal primary antibody (Spring Bioscience).The sections were then treated with goat anti-rabbit alkaline phosphatase labelled secondary antibodies and goat anti-mouse peroxidase labelled secondary antibodies respectively. DAB (3–3’diaminobenzidine) and fast red were used as chromogens for subsequent reaction detection. Tissue sections were counter stained with haemotoxylin, and mounted with DPX (Distrene plasticizer xylene). Double Immunostained slides were examined using Olympus^®^BX 50 optical microscope by gastrointestinal pathologist blinded to biopsy categories. Image morphometry was done using Image proplus 6.0 software (Media Cybernetics Inc., USA). A cool SNAP-PRO of color (Media Cybernetics Inc., USA) CCD camera was used to take photomicrographs of at least 5 representative areas by using 40 X objective only at 400 magnification and the digitized sections were then stitched. After staining, the CD4+ cells showed brown positivity and the FoxP3 cells showed red positivity. The cells which showed both FOXP3^+^CD4^+^ dual positivity, showed mixed reddish-brown colour. The number of these dual positive cells were then calculated, in respect to the total CD4^+^ T cell population, and the frequency of the FOXP3^+^CD4^+^ T cells were calculated in tissue sections. (Fig 1)

#### mRNA expression by real rime PCR (qPCR) in blood and colonic biopsies

RNA was extracted from collected tissue samples using the RNeasy commercial kit (Qiagen,USA) and from whole blood using TRIZOL reagent (Sigma, USA). RNA was reverse transcribed to cDNA using Superscript III reverse transcriptase enzyme (Invitrogen, USA). The mRNA expression of the target genes were quantified on Mx3000P qPCR thermal cycler. Target genes, *FOXP3*: *5’ CCACTGGTTCACACGCATGTT 3’* *and* *3’ CTTGCGGAACTCCAGCTCATC 3’* were amplified along with housekeeping genes, *CD4*: *5’ CCTCCTGCTTTTCATTGGGCTAG 3’ and 3’* TGAGGACACTGGCAGGTCTTCT 5’ and *GAPDH*: *5’ GCTCCTCCTGTTCGACAGTCA 3’* *and* *3’ GCAACAATATCCACTTTACCAG 5’* using SYBR Green chemistry. Threshold cycle (Ct) versus logarithms of cDNA concentration input were plotted to calculate slope and corresponding real time PCR efficiencies were calculated from given slopes in Maxpro software. Further, relative gene expression by qPCR was calculated for all the samples (blood and biopsy) with respect to study controls using REST 2009 software [Relative Expression Software Tool] [22].

### Statistical analysis

Continuous variables were expressed as mean ± SD or median [Inter-quartile range (IQR)] and categorical variables were expressed as percentage. Non parametric tests along with Post hoc correction test was used for multiple comparisons between CD and ITB groups. Analysis for differential pattern of CD4^+^FOXP3^+^ dual positive cell count in biopsy samples of CD and ITB was done using Mann-Whitney U-test for two group comparison and Dunn's Multiple Comparison Test used for all groups’ comparison. Receiver operating characteristics (ROC) curve was constructed to determine area under curve for FOXP3^+^ cells in peripheral blood. Statistical analysis was done using IBM SPSS Statistics 19.0 version and a p value <0.05 was considered as statistically significant.

## Results

### Baseline and demographic characteristics

124 patients were recruited in the study: 32 CD patients (19 in relapse, 13 in remission), 21 ITB patients (16 Pre-ATT, 5 Post ATT), 38 UC patients (18 in relapse, 20 in remission) and 33 controls. Baseline characteristics of controls, CD, UC and ITB patients are shown in Tables 1 and 2. No difference in mean age and gender distribution was observed between the four groups.

**Table 1:** Baseline characteristics of patients and controls.

		CD (n = 32)	ITB (n = 16 pre ATT)	UC (n = 38)	Controls (n = 33)	P value
Age (yrs)	Mean±SD	39.06+ 15.7				0.365
	Range	(18–66)	(18–70)	(25–65)	(18–61)	
Gender	Males (%)	14 (43.7%)	11(68.7%)	25(65.7%)	17 (51.5%)	0.128

**Table 2:** Clinical characteristics of patients with intestinal TB and Crohn’s disease.

		CD (n = 32)	ITB (n = 16)
Disease Location (n)	Terminal Ileum	0	0
	Colonic	17 (53.1%)	2(12.5%)
	Ileocolonic	15 (46.9%)	14(87.5%)
Disease Activity (n)	Remission	13(40.6%)	Active disease (pre ATT): 16(100%)
	Mild Relapse	10(31.2%)	
	Moderate to severe Relapse	9(28.1%)	
Drug therapy at enrollment (n)	5ASA	25 (78.2%)	-
	Azathioprine	7 (21.8%)	-
	Corticosteroids	-	-
	No drugs	-	16(100%)
Hemoglobin (gm/dl) (mean±SD)		11.03±2.3	11.04±3.4
ESR (mm) (mean±SD)		47±26.7	44±28
Platelets /mm^3^ (mean±SD)		317±156	217±108
Serum albumin (gm/dl) (mean±SD)		4.03±1.37	3.8±1.2

### Elevated FOXP3^+^ T cells in peripheral blood of ITB patients

**Fig 2:**
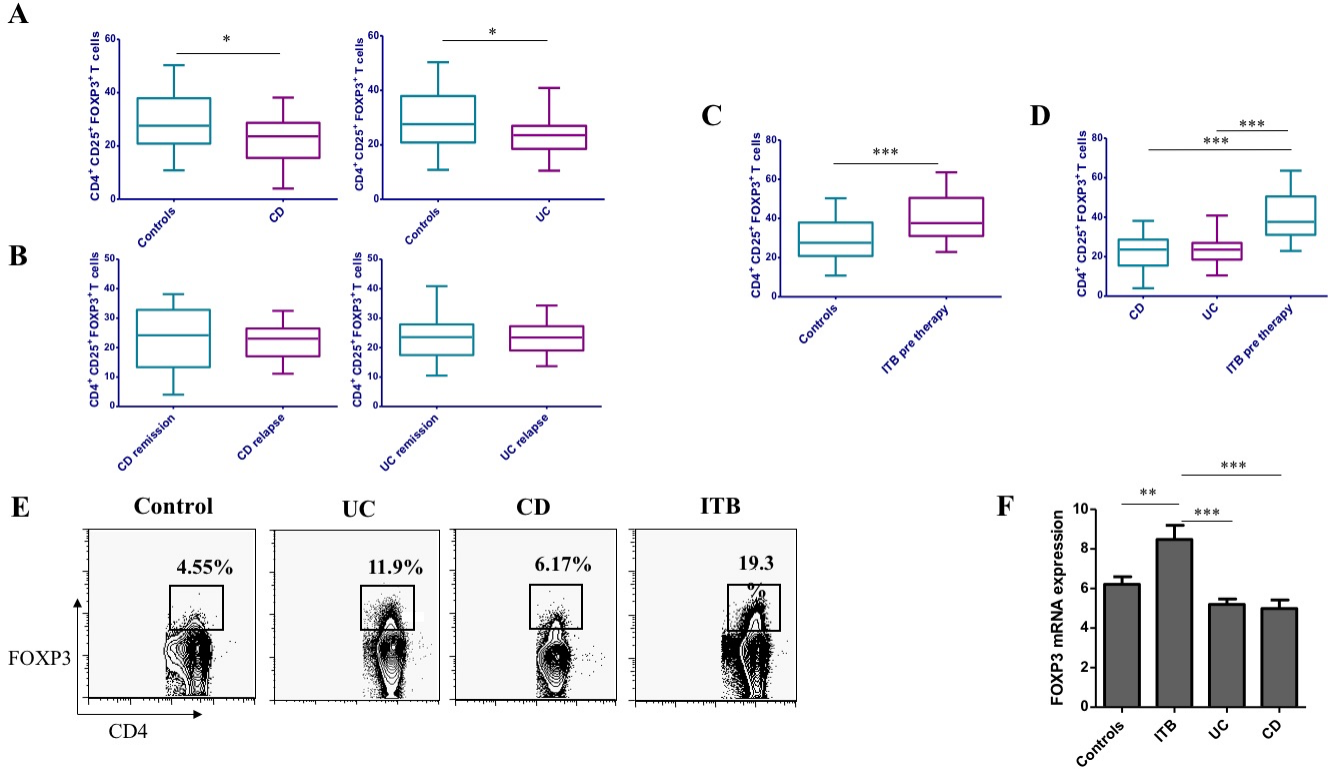
Comparison of peripheral CD4^+^CD25^+^ CD127^-^ FOXP3^+^ T cells are increased in ITB patients. Intracellular expression of FOXP3 gated on CD4^+^CD25^+^ CD127^-^ T cells were analysed by flow cytometry in (A) Controls, CD and UC populations (B) CD and UC in remission and relapse (C) Controls and ITB pre-therapy patients and (D)CD, UC and ITB pretherapy patients (E) Frequency of CD4^+^CD25^-^FOXP3^+^ T cells analysed by Flow cytometry and (F) Total FOXP3 mRNA expression were evaluated in Controls, CD, UC and ITB pre-therapy patients. * P<0.05, ** P<0.001, *** P<0.0001.

The frequency of CD4^+^CD25^+^ CD127^-^ FOXP3^+^ T cells in peripheral blood was significantly lower in CD [23.6% (15.5%–28.7%)] and UC patients [23.5% (18.5% -27.0%)] as compared to controls [27.6% (20.9% –37.9%), p = 0.0319, p = 0.027] (Fig 2A). Also, the disease activity status did not affect the FOXP3 cell frequency in CD and UC patients, as it was similar in patients with active disease and remission (Fig 2B). We also measured T reg frequency in another subset of eight naïve untreated CD patients and median frequency of T-reg cells in this cohort [23% (11.1% - 33.3%)], was similar to the results of previous cohort.

However, the FOXP3 cell frequency was significantly higher in ITB pre-therapy group as compared to controls (37.6% (31–50.5%) vs 27.6% (20.9–37.9%), p = 0.003) (Fig 2C), CD and UC patients (Fig 2D). Moreover, the FOXP3^+^ cell frequency came down significantly following antitubercular therapy (n = 5, p = 0.043), while no difference between ITB post therapy group and controls was observed (Table 3). FOXP3 mRNA expression in peripheral blood was also up-regulated in ITB (pre ATT, n = 12) as compared to CD patients (n = 20) by a mean factor of 2.47 (S.E. range 0.486–20.041, P = 0.037) (Fig 2F).

**Table 3:** Peripheral blood frequency of FOXP3 cells in various disease groups and controls.

Category	Number	% of FOXP3^+^ cells		P value*
		Median	IQR	
Controls	33	27.6	20.9–37.9	
Crohn’s Disease (Total)	32	23.6	15.5–28.7	0.0319
Crohn’s Disease (Relapse)	19	23.0	17.4–26.5	0.039
Crohn’s Disease (Remission)	13	24.1	13.3–32.8	0.176
ITB pre ATT	16	37.6	31.0–50.5	0.003
ITB post ATT	05	26.5	19.2–30.0	0.463
Ulcerative colitis (Total)	38	23.5	18.5–27.0	0.027
Ulcerative colitis (Relapse)	18	23.4	19.0–27.2	0.067
Ulcerative colitis (Remission)	20	23.5	17.5–27.9	0.069

* p values are in comparison with controls

We also determined the CD4^+^CD25^-^FOXP3^+^ T cells in the peripheral blood of controls, ITB and IBD patients. Similar findings were observed, where the CD25^-^FOXP3^+^ T cells were increased in ITB patients as compared to controls, CD and UC patients. Similar to its CD25^+^ counterpart, the CD25^-^ FOXP3^+^ T cells were significantly increased in ITB patients as compared to IBD (UC and CD) patients (Fig 2E).

### ITB patients have increased colonic CD4^+^ FOXP3^+^ cells

**Fig 3:**
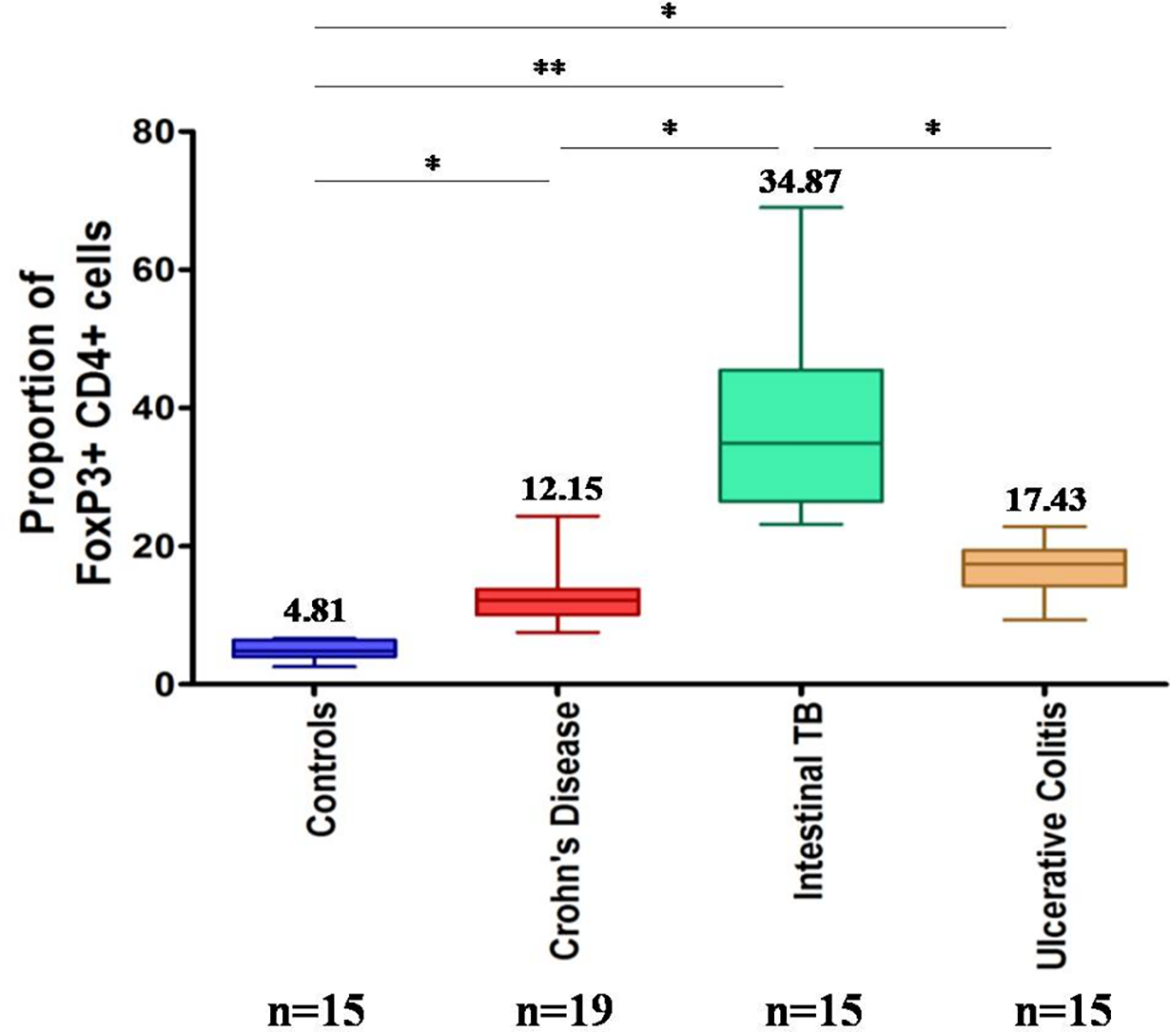
Comparison of colonic CD4^+^FOXP3^+^ T cells in controls, CD, UC and ITB patients as assessed by immunohistochemistry (IHC). * P<0.05, ** P<0.001, *** P<0.0001.

Next, we wanted to determine that like peripheral blood whether there are any differences in the colonic FOXP3 frequency in these patients. CD4^+^FOXP3^+^ cells as seen on immunohistochemistry of colonic biopsies, were significantly upregulated in ITB [34.9% vs 4.8%, p<0.001], CD (12.2% vs 4.8%, p<0.05) and UC patients [17.43% vs 4.81%, p<0.05] as compared to controls. Further, in the patient population, the FOXP3 frequency was observed to be significantly higher in ITB patients as compared to CD [34.9% vs 12.2%, p<0.05] and UC patients [34.9% vs 17%, p = <0.05] (Fig 3). In colonic biopsies, FOXP3 mRNA expression was also up regulated in ITB (in comparison to CD) by a mean factor of 2.235 (S.E. range is 0.792–6.408 p = 0.025).

### Peripheral FOXP3^+^ T cells as an immunological marker in differentiating ITB from CD

**Fig 4:**
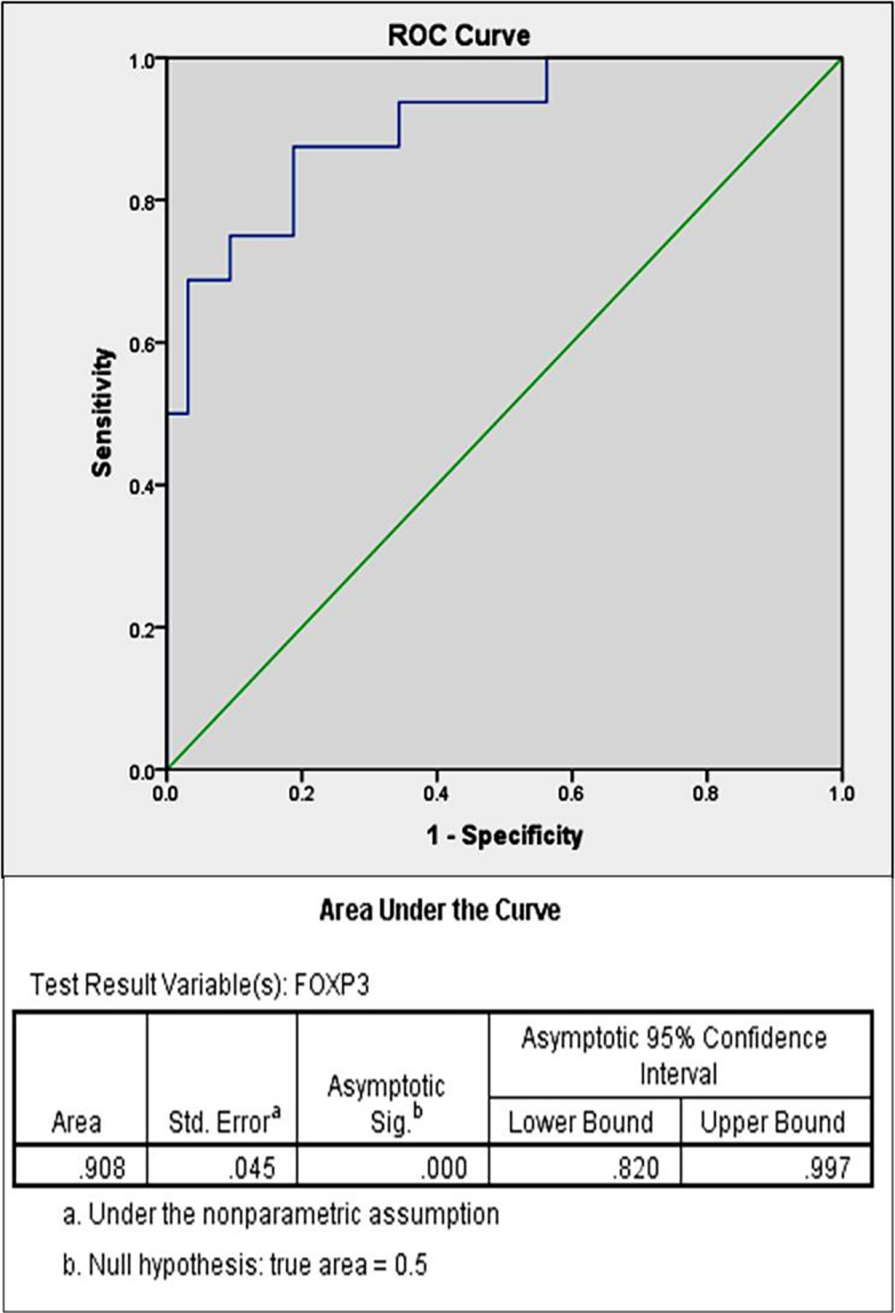
Receiver operating characteristics (ROC) curve plotted according to percentage of CD4^+^CD25^+^ CD127^-^ FOXP3^+^ cells from blood samples of ITB and CD patients.

To predict the cut off frequency of peripheral CD4^+^ CD25^+^ CD127^-^ FOXP3^+^ cells for differentiating between ITB and CD, ROC curve was drawn which revealed an area under the curve (AUC) of 0.908 (95%CI: 0.82–0.97, p<0.001). According to the coordinates of ROC curve, a cut off value of 32.54% FOXP3 cells in peripheral blood could predict a diagnosis of ITB with a sensitivity of 75% and specificity of 90.6% (Fig 4). Increasing the cut off value to 34.55%, decreased the sensitivity to 68.8% while increasing the specificity to 96.9%. Further increasing the cut off value to 38.9% decreased the sensitivity to 50% while increasing the specificity to 100%. Therefore, a cut off value of 32.54% had the highest diagnostic accuracy to distinguish ITB from CD.

## Discussion

Distinguishing ITB from CD in endemic areas for TB is challenging. Both ITB and CD are clinically and endoscopically very similar on appearance. To distinguish them, often ATT therapy is given which may lead to a significant time lag in achieving correct diagnosis and as a result delay in initiation of therapy for CD [10]. Therefore, there is a need for a diagnostic biomarker, which is easy to obtain for analysis, the results are available in a short period and have a high diagnostic accuracy.

This is the first such comparative study where FOXP3^+^ T cell frequency and expression was evaluated in peripheral blood as well as colonic biopsies in ITB as well as CD patients. The main findings of the study were that, during active infection, peripheral and colonic FOXP3 expression was significantly raised in both the Treg counterparts (CD25^+^ and CD25^-^) in ITB patients as compared to CD as well as UC patients.

There is a lot of discrepancy in the literature on the frequency and role of Tregs in IBD patients. Saruta et al [23] has reported an increase in peripheral FOXP3^+^ T cells in CD patients. On the contrary, Maul et al. have shown fewer Tregs in active CD than in inactive disease [24]. Similarly, other studies have also reported a decreased Treg frequency in peripheral blood of IBD patients [25,12]. In our study, in peripheral blood, we observed a decreased Treg frequency in IBD patients with active disease activity as compared to controls. In contrast to the results from studies of peripheral blood, observations in the murine gut consistently show an increase in the percentage of FOXP3^+^ cells in inflamed lamina propria and in mesenteric lymph nodes, particularly in and near inflamed tissue [26]. Like our findings that FOXP3^+^ cells and mRNA expression increase in inflamed region, there are other human studies too that report an increase in Treg numbers in IBD patients [27,28]. During inflammation, the FOXP3^+^ T cells rise in order to counterbalance the T effector responses but their inability to abrogate the intestinal inflammation suggests that either they have lost their suppressive capability [27] or they have gained the potential to differentiate into Th17 cells [29].However there is a lot of discrepant data with another study by Makita et al stating that the functional studies of Tregs isolated from IBD patients have similar suppressive capability as the Tregs isolated from control individuals [30]. Therefore, the role of Tregs in IBD still warrants a lot more investigation.

Despite of a wide range of literature available on the role of FOXP3 in IBD, there is scarce data available on its role in ITB. FOXP3 has been noted to be increased in the peripheral blood and sites of infection in patients with pulmonary TB [31]. Therefore, we hypothesized that FOXP3 might also play a role in ITB. For this we did a pilot study where CD4^+^ FOXP3^+^ T cells were observed to be increased in mucosal biopsies of CD patients and controls [16]. Taking this lead, the present prospective study was conducted with a larger sample size to investigate whether FOXP3 can be used as a biomarker to differentiate ITB from CD.

The key finding in this study was a higher frequency of FOXP3^+^ T cells in PBMCs as well as inflamed colonic mucosa of ITB patients with respect to controls as well as CD patients. This is the first study, which has looked at FOXP3^+^ T cell frequency in ITB patients. In addition to their role in immunological tolerance, FOXP3^+^ T cells play a crucial role in regulating the immune response to pathogens [32,33]. In concordance with the studies in pulmonary TB patients, our study suggests that there is an enrichment of FOXP3^+^ T cells in PBMCs as well as colonic tissue of ITB patients even without concomitant pulmonary TB. Further the CD patients showed an increase in colonic Tregs accompanied by a decrease in peripheral blood thereby suggesting a possible sequestration of peripheral Tregs in areas of active inflammation. Another interesting phenomenon, which we have demonstrated is significant reduction in FOXP3^+^ T cell frequency in PBMCs of patients with ITB following completion of ATT which verifies the expansion of FOXP3^+^ T cells during active infection. We have also shown that CD4^+^CD25^+^ FOXP3^+^ T cell frequency in PBMCs when plotted on a ROC curve for ITB and CD patients had a highly significant area under the curve [AUC = 0.908, p = 0.00, 95% CI = 0.82–0.99]. Patients with a diagnostic dilemma of CD or ITB with a cut off value of greater than 32.5% FOXP3^+^ cells were predicted to have ITB with a specificity of 90.6%.

We have previously shown that FOXP3 expression was upregulated in colonic biopsies but there was no difference in the serum of ITB as compared to CD patients [16]. This discrepancy could be due to the small sample size (4-CD, 5-ITB, 4-controls) of our previous study. On the other hand, the major strength of our study was evaluation of a prospective large cohort of IBD and ITB patients for FOXP3^+^ T cell expression in both blood and colonic mucosa and further use of this information for identifying FOXP3 as an immunological biomarker to differentiate ITB from CD. The major limitation of our study was to include CD patients who were already receiving treatment. Recently diagnosed IBD or treatment naïve cases would have probably reflected a more accurate cytokine environment. Secondly, ITB patients were included prior to the initiation of ATT therapy. Except five patients (post ATT therapy), most of the patients could not be followed up or refused consent to give repeat samples at the end of therapy.

In conclusion, phenotypic enumeration of peripheral CD4^+^CD25^+^FOXP3^+^ T cells can be a potential biomarker to differentiate between ITB and CD, as it requires a blood sample, which makes it a least invasive test and the results can be obtained on the same day with a high diagnostic specificity.
